# Supplementary material for: Collaborative intelligence in AI: Evaluating the performance of a council of AIs on the USMLE
Source: PLOS Digit Health. 2025 Oct 9;4(10):e0000787. doi: 10.1371/journal.pdig.0000787 (PMC12510544; doi:10.1371/journal.pdig.0000787)
Supplement: S2 File — (ZIP) [file pdig.0000787.s002.zip › S2 Data Raw data files containing unprocessed question inputs and LLM outputs including transcripts of deliberations/STEP3_Question-37-1_Type-MC-NJ.docx]

**Facilitator** **[Round 1]:** A 70-year-old man is brought to the emergency department by his son because of a 2-day history of right upper quadrant abdominal pain, chills, and confusion. He has vomited twice during this time despite decreased food intake. The patient has hypertension controlled with hydrochlorothiazide. Medical and surgical history is otherwise unremarkable. Vital signs are temperature 40.2°C (104.4°F), pulse 110/min, respirations 18/min, and blood pressure 100/60 mm Hg. The patient appears seriously ill without evidence of jaundice. Abdominal examination discloses tenderness to palpation over the liver without guarding. Results of laboratory studies are shown: Serum: Amylase 350 U/L Bilirubin total 2.1 mg/dL Blood: WBC 16,500/mm3 with prominent immature forms Neutrophils, segmented 42% total Neutrophils, bands 25% Serum urea nitrogen and creatinine concentrations are within the reference ranges, as are results of liver function tests. Ultrasonography of the abdomen shows multiple stones in the gallbladder and a common bile duct measuring 9 mm in diameter. Intravenous fluids and a broad-spectrum antibiotic are administered. Which of the following is the most likely diagnosis? (A) Acute cholecystitis (B) Acute pancreatitis (C) Cholangitis (D) Hepatitis (E) Liver abscess

**AI_C:** RESTATING THE QUESTION: An elderly man is presented with symptoms including high fever, rapid pulse, low blood pressure, abdominal pain, chills, confusion, and a history of hypertension. There are multiple gallstones, a dilated common bile duct, leukocytosis with left shift, and a high serum amylase level and slightly raised bilirubin level. From the available options (Acute cholecystitis, Acute pancreatitis, Cholangitis, Hepatitis, Liver abscess), we have to diagnose his condition. PLANNING: Assumptions that we can consider— severe infection considering the patient's age, clinical presentation, and laboratory findings (prominent immature forms of WBCs signify systemic inflammation), obstruction by gallstones causing inflammation and infection (shown by ultrasound and raised amylase, respectively), or a liver-specific disease (hepatitis or liver abscess). We should start by ruling out the liver diseases as his liver function tests are within normal range. CLINICAL REASONING: The patient's symptoms, along with abdominal tenderness over the liver, may suggest a hepatobiliary disease. His lab findings offer a few key points: evidence of gallstones, dilated common bile duct, raised amylase levels, and a relative neutrophilia suggesting a systemic inflammatory response. Hepatitis and liver abscesses could cause similar symptoms, but the absence of abnormal liver function tests makes these less likely. The most predictive factor here might be the patient's lack of jaundice; this tends to occur with hepatocellular diseases like hepatitis or liver abscesses. Instead, these symptoms may align with either cholangitis or acute cholecystitis, conditions caused by gallstones. Although the elevation of amylase can often be associated with pancreatitis, the absence of significant upper quadrant pain radiating to back and a normal serum urea nitrogen and creatinine (typically raised in pancreatitis due to renal involvement by inflammation) makes pancreatitis less probable. CONCLUDING: Thus, every clue thus far is directing us toward a gallstone disease leading to systemic inflammation and infection not localized to liver. RESPONSE: Therefore, in the context of the patient's clinical picture, laboratory findings, ultrasound results, and his negative liver function tests, the most likely diagnosis is (C) Cholangitis. This is a condition characterized by inflammation of the bile ducts due to bacterial infection, often precipitated by obstruction from gallstones. This would explain the patient's systemic symptoms, as well as his localized abdominal pain, without signs of jaundice.

**AI_D:** (1) RESTATING THE QUESTION: The presented case is about a 70-year-old man with a history of hypertension, who presents with a 2-day history of fever, confusion, chills, vomiting, right upper quadrant abdominal pain, and tenderness on palpation of the liver. His lab results show elevated WBCs with a left shift, mildly elevated bilirubin and amylase, and normal liver function tests. Furthermore, ultrasonography shows gallstones and an enlarged common bile duct. The question is asking for the most probable diagnosis among Acute cholecystitis, Acute pancreatitis, Cholangitis, Hepatitis, or a Liver abscess. (2) PLANNING: Initially, it's essential to realize that the elevated WBC count along with the left shift (presence of immature neutrophils) indicates an ongoing infection. The presence of gallstones and an enlarged common bile duct could suggest a gallbladder or bile duct related disorder. However, there is no jaundice, which is commonly present in severe forms of biliary tract diseases. Comparing the symptoms and results against the provided potential diagnoses, Cholangitis seems to be the most plausible. (3) CLINICAL REASONING: The Charcot's triad: right upper quadrant pain, fever and jaundice are common in cholangitis. However, in this case, jaundice is absent. It's important to note that one component of the triad may be absent in about 50% of cases. In addition to Charcot’s triad, if altered mental status and septic shock, represented by hypotension, are present, this is referred to as Reynold's pentad. Reynold's pentad is indicative of severe cholangitis, as in this case, which explains patient’s confusion and chills, along with tachycardia. The tender hepatomegaly can be present in both cholangitis and acute cholecystitis, but it is more characteristic to cholangitis. Elevated WBC counts with a left shift, indicating prominent immature forms, also assert the possibility of a systemic infection. (4) CONCLUDING: Putting all the pieces together, clinical features such as right upper quadrant abdominal pain, high fever, a change in mental status (confusion), elevated WBC count and prominent immature neutrophils indicating an infection, along with absence of jaundice, and ultrasound findings of multiple gallstones and a widened common bile duct, the most likely diagnosis is cholangitis, despite the absence of jaundice that is commonly associated with this condition. (5) RESPONSE: Therefore, based on the patient's presenting symptoms, lab findings, and ultrasound results, the most likely diagnosis is (C) Cholangitis.

**AI_B:** RESTATING THE QUESTION: This query essentially concerns a geriatric patient presenting with symptoms of right upper quadrant abdominal pain, chills, and confusion, coupled with clinical findings. These include elevated body temperature, tachycardia, hypotension, abdominal tenderness, elevated WBC with immature forms and bands, increased serum amylase, mildly raised bilirubin, and an abdomen ultrasound revealing gallstones and dilated common bile duct. We are being asked to determine the most appropriate diagnosis from the choices given. PLANNING: The key assumptions are that the symptoms, signs, and investigation findings correlated with the patient's clinical state are indicative of one of the diseases listed: Acute cholecystitis, Acute pancreatitis, Cholangitis, Hepatitis, and Liver abscess. For a better assumption, we could consider that the patient might be suffering from a disease that correlates with all of these symptoms and signs, and the predominant clinical feature tends to guide us towards one diagnosis over others. CLINICAL REASONING: When considering these assumptions, it's essential to apply clinical reasoning. The patient’s presenting symptoms, elevated temperature, and laboratory data showing leukocytosis with left shift (immature forms) indicate an ongoing systemic infectious process. Right upper quadrant pain along with hepatomegaly and chills, without any apparent signs of jaundice or guarding, lead us to think about an intraabdominal infectious process rather than an inflammatory one. The elevated serum amylase could be misleading towards pancreatitis, but the absence of symptoms typical of pancreatitis and isolated mildly elevated amylase without accompanying lipase weight against it. The ultrasound findings of gallstones in the gallbladder and dilated common bile duct could relate to the obstructive pathology associated with infection. CONCLUDING: It's evident from the overall clinical picture of systemic signs of infection (fever, leukocytosis), right upper quadrant pain, non-jaundiced status, positive abdominal findings, and radiological evidence of gallstones and dilated bile ducts, that the most likely diagnosis would tend to be a hepatobiliary tract infection rather than a purely inflammatory or abscess-forming process. RESPONSE: The most likely diagnosis in this clinical scenario is (C) Cholangitis.

**AI_E:** RESTATING THE QUESTION: The case in question involves a 70-year-old male patient who has been experiencing right upper quadrant abdominal pain, chills, and confusion for the past two days. Accompanying symptoms include vomiting and an apparent decrease in food intake. This patient's hypertension is currently under control thanks to hydrochlorothiazide. Despite his serious condition, he does not exhibit signs of jaundice, although he does present with liver tenderness upon palpation. Laboratory tests have been conducted, yielding results that indicate an elevated WBC count with a significant presence of premature forms, an increase in total bilirubin value, and an increase in amylase concentration. However, serum urea nitrogen, creatinine, and liver function test results appear normal. Abdominal ultrasonography has revealed multiple gallstones as well as an abnormally enlarged common bile duct. After assessing the situation, healthcare professionals have opted for an IV fluid and broad-spectrum antibiotic regimen. We are tasked with pinpointing the most probable diagnosis out of the following options: acute cholecystitis, acute pancreatitis, cholangitis, hepatitis, or a liver abscess. PLANNING: Based on the information provided, we may make the following assumptions and questions. First, the presence of right upper quadrant pain along with fever and chills could indicate an inflammatory process, possibly involving gallbladder pathology. Could it be acute cholecystitis? Alternatively, elevated levels of serum amylase may suggest acute pancreatitis. Or could the patient's symptoms and imaging findings of stones in the gallbladder and an enlarged common bile duct suggest cholangitis? Also, consider hepatitis or liver abscess as a possible explanation for this patient's symptoms. However, this patient does not exhibit jaundice, a common manifestation of hepatitis, and while liver abscess may present with a similar clinical picture, there is no clear supporting evidence in laboratory or imaging findings to confirm this. Thus, considering the clinical presentation, lab findings, and ultrasonography results, we should prioritize the assumption of cholangitis. CLINICAL REASONING: Cholangitis is an infection of the bile duct, usually occurring due to an obstruction, which results in a bacterial infection ascend from the intestines into the bile duct. This condition, known as Charcot's triad, typically presents with the symptoms of abdominal pain, fever, and jaundice. However, the absence of jaundice does not definitively rule out cholangitis, especially when other strong indications are present, such as leukocytosis which signals an ongoing inflammatory response. Further, the bile duct dilation and presence of gallstones noted on the ultrasound could be the cause of obstruction leading to infection. Elevated serum amylase levels may also be due to the inflammation or obstruction of the biliary tract and not necessarily indicative of pancreatitis alone, especially given the absence of severe epigastric pain which is a hallmark symptom of acute pancreatitis. CONCLUDING: With the combination of severe right upper quadrant abdominal pain, chills, and confusion which may suggest systemic effects of the infection, along with imaging findings of an enlarged bile duct and gallstones, we should consider cholangitis as the prime diagnosis. This is further supported by the administration of broad-spectrum antibiotics, treatment common in cholangitis to cover gram-negative enteric bacteria including E. coli, Klebsiella spp., and Enterobacter spp., commonly implicated in this condition. RESPONSE: Therefore, based on the available clinical presentation, investigation findings, and management, the most likely diagnosis in this 70-year-old patient is (C) Cholangitis.

**AI_A:** RESTATING THE QUESTION: A 70-year-old man with a history of hypertension is seeking medical help due to two days of severe abdominal pain on the right upper quadrant, fever with chills, and states of confusion. He also exhibits loss of appetite and has vomited twice so far. Upon examination, the man appears seriously unwell, but shows no signs of jaundice. Abdominal palpation reveals pain over the liver area. His lab results disclose increased serum amylase, slightly elevated total bilirubin, and a high white blood cell count with a considerable degree of immature forms and banded neutrophils. Other lab parameters, including liver function tests, are within the normal range. An ultrasound of the abdomen uncovers multiple gallstones and an expanded common bile duct. The patient has been started on IV fluids and a broad-spectrum antibiotic. We are tasked to diagnose the most plausible condition from options: Acute cholecystitis, Acute pancreatitis, Cholangitis, Hepatitis, and Liver abscess. PLANNING: Let's explore each assumption individually: (A) Acute cholecystitis generally presents with a severe, often constant, pain in the right upper quadrant or epigastric area. Fever, nausea, vomiting, and a mild increase in white blood cells are other common symptoms, but this condition is not usually associated with confusion which has also been reported as part of our patient's presentation. (B) Acute pancreatitis often presents with upper abdominal pain that often radiates to the back, accompanied by increased levels of pancreatic enzymes (e.g. amylase, lipase) in the blood. Our patient does have elevated amylase, however, pancreatitis is generally not associated with the dilation of the common bile duct that our patient's ultrasound has shown. (C) Cholangitis typically presents with jaundice, fever with chills (Charcot's triad), and right upper quadrant pain pointed out in this case. Additionally, altered mental status and septic shock (known as Reynold's pentad) can also occur, which could explain the present confusion in our patient. (D) Hepatitis or inflammation of the liver, might cause right upper quadrant pain, but the patient's liver function tests are within normal ranges which contradict this assumption. (E) Liver abscess could potentially explain fever, right upper quadrant pain and altered mental status (confusion). However, the lack of jaundice which frequently accompanies this condition makes it a less likely diagnosis. Considering the presenting symptoms, lab results, and imaging findings, the most clinically prioritized assumption to pursue further here is likely to be "Cholangitis." CLINICAL REASONING: Cholangitis develops due to a bacterial infection in the bile duct, usually subsequent to obstruction. In this case, the obstruction could likely have happened due to gallstones identified in the ultrasound. An obstructed bile duct can foster bacterial growth leading to an infection - cholangitis. Systemic symptoms such as fever, chills, and confusion could arise from the spread of bacteria or their toxins beyond the bile duct into circulation. Lab findings align with this interpretation, showing an increased WBC count indicating a likely bacterial infection. Our patient's confusion can be explained by the sepsis resulting from this escalating infection. CONCLUDING: To conclude, our patient's clinical presentation of right upper quadrant pain, fever with chills, confusion, as well as, lab reports indicating elevated serum amylase, mild elevation of bilirubin, increased WBC count, and imaging showing multiple gallstones and dilated common bile duct, all pivot towards the most probable diagnosis of Cholangitis, a condition resulting from bacterial infection in a likely obstructed bile duct. RESPONSE: Hence, considering the presented symptoms, clinical signs, laboratory investigations, and the observation from abdominal ultrasonography, the most likely diagnosis for this 70-year-old patient seems to be "(C) Cholangitis".

**Facilitator** The consensus statement synthesizes the responses of AI_A, AI_B, AI_C, AI_D, and AI_E as follows: The case presents a 70-year-old man with a history of hypertension, who is experiencing severe right upper quadrant abdominal pain, fever with chills, and confusion. He also exhibits loss of appetite and has vomited twice. Despite appearing seriously unwell, he shows no signs of jaundice. His lab results reveal increased serum amylase, slightly elevated total bilirubin, and a high white blood cell count with a considerable degree of immature forms and banded neutrophils. An ultrasound of the abdomen uncovers multiple gallstones and an expanded common bile duct. Given the patient's symptoms, lab results, and imaging findings, the most plausible diagnosis is Cholangitis. This condition typically presents with right upper quadrant pain, fever, and jaundice (Charcot's triad), and in severe cases, altered mental status and septic shock (Reynold's pentad). However, the absence of jaundice does not definitively rule out cholangitis, especially when other strong indications are present. The patient's confusion could be explained by the sepsis resulting from this escalating infection. Cholangitis develops due to a bacterial infection in the bile duct, usually subsequent to obstruction. In this case, the obstruction could likely have happened due to gallstones identified in the ultrasound. An obstructed bile duct can foster bacterial growth leading to an infection - cholangitis. Systemic symptoms such as fever, chills, and confusion could arise from the spread of bacteria or their toxins beyond the bile duct into circulation. Therefore, considering the presented symptoms, clinical signs, laboratory investigations, and the observation from abdominal ultrasonography, the most likely diagnosis for this 70-year-old patient seems to be Cholangitis. This is a condition characterized by inflammation of the bile ducts due to bacterial infection, often precipitated by obstruction from gallstones. This would explain the patient's systemic symptoms, as well as his localized abdominal pain, without signs of jaundice.
